# Epidemiology of Paediatric Orthopaedic Trauma, Before, During, and After the Outbreak of COVID-19 Pandemic: An Observational Study From a Tertiary Referral Center in Wales

**DOI:** 10.7759/cureus.19253

**Published:** 2021-11-04

**Authors:** Sandeep Gokhale, Prashanth D'sa, Abdul Azeem Badurudeen, Eleanor Clare Carpenter

**Affiliations:** 1 Trauma and Orthopaedics, University Hospital of Wales, Cardiff, GBR; 2 Trauma and Orthopaedics, University hospital of Wales, Cardiff, GBR

**Keywords:** epidemiology, post-lockdown, lockdown, baseline, covid-19, paediatric trauma

## Abstract

Introduction

The outbreak of coronavirus disease 2019 (COVID-19) and the resultant lockdown has had a great impact on global healthcare. This observational study aimed to analyse the consequences of national lockdown on the epidemiology of significant paediatric orthopaedic trauma, presenting to a tertiary referral centre in Wales, during the COVID-19 pandemic in the United Kingdom.

Methods

Paediatric patients presenting with orthopaedic trauma, from March 2019 to July 2019 (baseline period), March 2020 to July 2020 (lockdown period), and March 2021 to July 2021 (post lockdown period), were identified and compared. Those aged less than 16 years, presenting with a significant orthopaedic injury, defined here for the study as, those requiring either manipulation under anaesthesia or, surgical intervention were included in this study.

Results

Mean age of children with significant orthopaedic injuries during the lockdown dropped significantly to 6.52 years in comparison to those during the baseline period (7.9 years), and post lockdown period (8.85 years). The incidence of outdoor injuries plummeted significantly from 64.71% in the baseline period, to 41.27% during the lockdown period, but rose to 80.65% in the post lockdown period. There was a 48% reduction in the number of children who required intervention in operating theatre during the lockdown period, in comparison to the other two study periods.

Conclusion

Our study reports a significant decrease in the incidence of paediatric orthopaedic trauma needing intervention in operating theatre during the lockdown period, with a significant rise in the incidence of domestic injuries, and relatively younger children sustaining these injuries. A public information campaign could help reduce the incidence of such domestic accidents during future lockdowns. We recommend improving awareness among parents, creating a safe indoor as well as outdoor environment to help reduce the incidence of fractures in the paediatric population. This will subsequently help in reducing the financial burden on an already stretched healthcare system.

## Introduction

The outbreak of coronavirus disease 2019 (COVID-19) caused by the severe acute respiratory syndrome coronavirus-2 (SARS CoV-2) has caused a global crisis affecting the frontline services, including the trauma and orthopaedics department [1-4]. Many countries affected by the pandemic resorted to a nationwide "lockdown". Similar lockdown measures were followed in the United Kingdom, which led to the closure of schools, sport activity centres and travel restrictions. These activities are known to be associated with paediatric traumatology [5]. This study aimed to investigate the impact of lockdown on the epidemiology of paediatric orthopaedic trauma requiring intervention in theatre and compare it with injuries presented in a corresponding time frame in the years before and after the pandemic. This study was conducted at a tertiary paediatric referral centre in Wales, UK.

## Materials and methods

This is an observational study looking at paediatric (age 0-16years) patients managed by the paediatric orthopaedic unit at a tertiary referral centre, during the lockdown period between March 2020 - July 2020. This cohort was compared with the children requiring treatment in the pre-pandemic period of similar duration (the baseline period, March 2019 - July 2019), and in the following year when there was no lockdown (the post lockdown period, March 2021 -July 2021). Children who had sustained a significant orthopaedic injury (fractures, soft tissue injury), defined here for the study as, one that required admission for management in operation theatre either by manipulation under anaesthetic, or surgical intervention, were included in the study. Exclusion criteria were those who had injuries requiring management by other surgical specialities and pathological fractures.

Data were collected from the centre’s paediatric trauma registry, on demographics, mechanism, location, nature of the injury, and the procedure undertaken. This study was registered and approved by the local research department as a service evaluation and did not need ethical committee approval.

Statistical analysis: All collected data were tabulated on Microsoft Excel (Microsoft Corporation, Redmond, Washington, USA), and analysed using simple descriptive statistics. Two-tailed Student’s t-tests were used to evaluate differences in continuous variables, while Fisher’s exact test or Chi-square test was used to evaluate differences in categorical data. Fisher’s exact test was used in proportion comparisons where values in any cells fell below 5. The Chi-square test was used in proportion comparisons where values in all cells were above 5. The one-way analysis of variance (ANOVA) was used to evaluate the differences between the means of three independent groups. Significance was defined at p≤0.05. Data were analysed using Statistical Product and Service Solutions package 20.0 (IBM SPSS, Armonk, New York, USA).

## Results

There was an overall 48% reduction in the number of patients who required intervention in operating theatre during lockdown period, in comparison to baseline, and post lockdown period. The mean age of children needing intervention in theatre during lockdown dropped significantly to 6.52 years in comparison to those during the baseline period of 7.9 years and rose to 8.85 years in the post lockdown period (see Table 1). Boys outnumbered girls during all the three-time periods (see Table 2). 

**Table 1:** Mean age and standard deviation in years of children in three study periods. n=number, SD=standard deviation.

Study group	Baseline	Lockdown	Post lockdown	p-value^*^
n	119	63	62	0.0041
Age (in years) Mean ± SD	7.91 ± 3.91	6.52 ± 3.70	8.85 ± 4.16	

**Table 2:** Gender distribution of children in the three study periods. n=number.

Gender	Baseline (n=119)	Lockdown (n=63)	Post lockdown (n=62)	p-value^*^
Male, n (%)	76 (63.87%)	36 (57.14%)	40 (64.52%)	0.6166
Female, n (%)	43 (36.13%)	27 (42.86%)	22 (35.48%)	

Incidence of outdoor injuries decreased significantly to 41.27% during the lockdown period, in comparison to 64.71% in the baseline period, but rose to 80.65% in the post lockdown period. There was a relative increase in the incidence of domestic injuries requiring intervention in theatre (see Table 3). Upper limb fractures remained the most common injury throughout all three study periods (72.2%, 63.8%, and 70.45%, respectively), with forearm fracture being the commonest injury overall. Incidence based on the type of injury/body part involved is detailed in Table 4. 

**Table 3:** Incidence of injuries based on the location of injury in the three study periods. n=number.

Location of injury	Baseline (n=119)	Lockdown (n=63)	Post lockdown (n=62)	p-value^*^
Indoor, n (%)	42 (35.29%)	37 (58.73%)	12 (19.35%)	0.000026
Outdoor, n (%)	77 (64.71%)	26 (41.27%)	50 (80.65%)	

**Table 4:** Incidence of Injury based on the region of body part involved in the three study periods n=number.

Injury region	Baseline (n=119), n (%)	Lockdown (n=63), n (%)	Post lockdown (n=62), n (%)
Clavicle	1 (0.84%)	0	0
Elbow	21 (17.65%)	5 (7.94%)	12 (19.35%)
Forearm	35 (29.41%)	15 (23.81%)	17 (27.42%)
Phalanx	3 (2.52%)	3 (4.76%)	2 (3.23%)
Nailbed	22 (18.49%)	13 (20.63%)	10 (16.13%)
Pubic Ramus	1 (0.84%)	0	0
Femur	6 (5.04%)	4 (6.35%)	3 (4.84%)
Tibia	8 (6.72%)	5 (7.94%)	3 (4.84%)
Ankle	8 (6.72%)	3 (4.76%)	7 (11.29%)
Navicular	0	1 (1.9%)	0
Soft tissue	17 (14.29%)	14 (22.22%)	8 (12.90%)
Meniscus	1 (0.84%)	0	0

Types of procedures carried out during the three study periods have been described in Table 5. 

**Table 5:** Procedures performed in the three study periods. MUA=manipulation under anaesthesia, ORIF=open reduction and internal fixation, n=number.

Procedure	Baseline (n=119), n (%)	Lockdown (n=63), n (%)	Post lockdown (n=62), n (%)	p-value^*^
MUA + Plaster	18 (15.13%)	15 (23.81%)	11 (17.74%)	0.034691
Pinning/ORIF	62 (52.10%)	18 (28.57%)	34 (54.84%)	
Nailbed repair	22 (18.49%)	13 (20.63%)	10 (16.13%)	
Wound Exploration, Washout and Closure	17 (14.29%)	17 (26.98%)	7 (11.29%)	

There was a slight increase in the incidence of trampoline-related injuries requiring intervention in theatre during the lockdown period (n=8, 12.70%) compared to the other two study periods (baseline n=8, 6.72%, post lockdown n=2, 3.23%, p = 0.1195), but this was not statistically significant.

## Discussion

Throughout the world, this pandemic has brought about major changes in protocols, and management strategies [6,7]. Meticulous strategies were outlined to manage preoperative, intraoperative, and postoperative periods [8]. Conservative/non-surgical treatments were favoured in some centres [9]. At our centre, we modified our approach according to the guidelines set by the British Orthopaedic Association [10]. All non-urgent, elective cases were deferred, and priority was given to the emergency/trauma cases. There was a tendency towards non-operative management of traumatic injuries, where possible, with the main aim of safely, effectively, and rapidly managing these paediatric trauma patients.

Our study shows a significant decrease (48%) in the number of children with orthopaedic injuries requiring intervention in theatre during the lockdown period. This effect was anticipated due to the national guidelines on social distancing measures, quarantining at home, and the resultant modification in people’s behaviour. Restrictions on travel and closure of sports centres, schools brought about a significant reduction in overall outdoor activities. Fear of acquiring COVID-19 infection by attending the hospital has also brought about a decrease in patients presenting to the emergency department to some extent [11]. A trend towards non-operative management of some injuries has also had an impact on these figures, however, cannot be accurately quantified. Similar changes in incidence have also been observed by other countries [1,12-13]. In Iran, Nabian et al., reported a 50% reduction of emergency patients, whereas Italy reported a 73-88% decrease in paediatric emergency attendances [1,14].

The mean age of children sustaining these injuries in our study decreased significantly from 7.9 years (baseline) to 6.52 years (lockdown). During the post lockdown period, the mean age climbed up to 8.85 years (p=0.004). Similarly, Bolzinger et al., reported a mean age of children sustaining an injury of 9.2 years during the baseline period in comparison to that during the lockdown of 7.9 years [6], and Bram et al., reported a mean age of 9.4 years, and 7.5 years respectively in these periods [15]. These suggest a general trend towards younger children sustaining injuries during the lockdown period. 

Recent studies have reported a decrease in the number of high energy fractures secondary to a lower incidence of road traffic accidents, outdoor injuries, and injuries related to sports during the COVID-19 outbreak [16]. A report by Christey et al., also found the incidence of injuries increased at the farm, at home, and decreased accidents elsewhere, an overall reduction in trauma patients attending the hospitals [12]. Similarly, we also noticed a change in trend concerning the location where the injuries occurred, which was most likely due to the national lockdown measures, and social distancing rules. We established an increase in the incidence of domestic accidents during the lockdown period (58.73%, p =0.00002), however, there was no increase in reported safeguarding issues. On the contrary, outdoor, and contact sports-related trauma had decreased significantly. After the lockdown was lifted, the trend of injury shifted to the outdoor setting once again, with a rebound increase to more than 80%. Palmer et al., describe ’lockdown fatigue’, the waning parental supervision as one of the causes for the increase in the number of domestic injuries among children [17]. 

Bolzinger et al. found a remarkable rise of trampoline-related injuries during lockdown [6]. We did notice a slight increase in the incidence of trampoline-related injuries requiring intervention in theatre during the lockdown period compared to the other two periods, however, this was not statistically significant (p = 0.1756). 

The predominance of injury among boys is demonstrated well in the literature [5,6,18,19]. In our study, injuries among girls increased from 36.13% (baseline) to 42.86% during the lockdown, however, returned to baseline figures (35.4%) post lockdown. These changes were, however, not statistically significant. The incidence of injury in boys remained dominant, occupying around 3/5th of the spot during all three study periods. 

For procedures performed in the operation theatre, open reduction with internal fixation (ORIF)/pinning was the commonest type of procedure performed during the baseline period (52.10%), soft tissue procedures such as wound exploration, washout, and closure were the least common ones (14.29%). In comparison, during the lockdown period, soft tissue procedures increased significantly (26.98%, p = 0.03). In the post lockdown period, there was a fall in soft tissue procedures to below the baseline figures (11.29%). This change in pattern, number of fractures, and the corresponding rise of soft tissue injury suggests that the lockdown was associated with change in overall pattern of injuries.

The strengths of this study include comparison groups for baseline and post lockdown periods. The limitations include data from a single centre, relative proportions are being compared and not the absolute numbers which show relative increase/decrease as per the comparison, the inherent bias of the observational study design, and do not account for confounding factors. However, this study provides valuable information on changes to the epidemiology of paediatric fractures to help guide resource management for future pandemics that we may encounter. To the best of our knowledge, this is the first report that observes, and compares the incidences of paediatric trauma requiring intervention in theatre in three different timelines; before, during, and after the lockdown imposed due to the COVID-19 pandemic.

## Conclusions

Our study shows a 48% decrease in the frequency of paediatric orthopaedic trauma needing intervention in operating theatre during the lockdown period. This might be due to the fact that modified approach was followed as per the guidelines by the British Orthopedic Association, since the selection/operative criteria has changed, the proportion of cases operated might be less. Further studies are required to exactly quantify the change in the incidence of paediatric trauma once the patient management criteria are back to normal. There was a relative rise in the incidence of domestic injuries requiring intervention in theatre, and more younger children were sustaining these injuries. This report should help the paediatric scientific society, and healthcare bodies, to set up appropriate guidelines, and strategies for the management of paediatric orthopaedic cases. A public information campaign could help reduce the incidence of such domestic accidents during future lockdowns. We recommend improving awareness among parents, creating a safe indoor as well as the outdoor environment in recreational places to help reduce the incidence of fractures in the paediatric population. This will subsequently help in reducing the financial burden on an already stretched healthcare system.
